# The relationship between psychological capital and work engagement of kindergarten teachers: A latent profile analysis

**DOI:** 10.3389/fpsyg.2023.1084836

**Published:** 2023-03-09

**Authors:** You Gao, Yaping Yue, Xiaomin Li

**Affiliations:** ^1^Faculty of Education, Beijing Normal University, Beijing, China; ^2^Henan Preschool Education Research Center, Faculty of Education, Henan University, Kaifeng, China

**Keywords:** kindergarten teacher, psychological capital, work engagement, relationship, latent profile analysis

## Abstract

Although the importance of psychological capital has been firmly supported by prior studies, the question of whether certain subgroups exist and how these various subgroups affect work engagement differentially remains under-explored. To gain an in-depth understanding of this problem, the present study conducted a person-centered method (latent profile analysis) to identify subgroups and then explore the relationship between psychological capital subgroups and work engagement. The study participants were kindergarten teachers in China (*n* = 2,790). The results showed that psychological capital was divided into three latent profiles: “rich type” (43.2% of the sample) “medium type” (46.3%), and “poor type” (10.5%). Compared to the other two types, the teachers with high psychological capital returned higher work engagement scores. Meanwhile, there was a significant difference among the three identified profiles regarding the location of the kindergarten, the type of kindergarten, and the teaching experience. The group owning more psychological capital tended to have more teaching experience, come from a developed area, and work in a public kindergarten. And after controlling the influence of the type of kindergarten, the location of kindergarten, and the teaching experience, the psychological capital of kindergarten teachers was still an important factor that affected teachers’ work engagement.

## Introduction

The emphasis of Chinese government on the development of preschool education and the change in China’s regulations of preschool education in recent years has pushed a more complete preschool education system in spite of causing work stress to preschool teachers. Taken together, with respect to the current educational environment, in the site of preschool education, in addition to taking good care of and teaching children in their class, providing them with warm, nurturing and learning experience so as to lay the foundation for their future social, emotional and academic success (Coplan et al., 2015), kindergarten teachers have to treat heavy and trifle administrative affairs and react to each change in preschool education policies internally. On the other hand, they have to experience pressure from the parents for parent-teacher communication (Hung, 2012). Thus, kindergarten teachers are trapped in conditions that need to endure tremendous pressure, which is not conducive to improving their work engagement and thus affect the quality of preschool education. In the process of personal work and development, psychological capital is a crucially important positive psychological factor (Luthans et al., 2007a). Studies have shown that psychological capital can increase a person’s sense of identity, belonging, and mission inside an organization (Wang and Li, 2011), lower the percentage of employees who leave (Avey et al., 2006), and significantly improve the quality of work (Allameh et al., 2018). In a high-pressure and challenging job environment, if kindergarten teachers demonstrate high psychological capital, it will have a significant impact on children’s psychological growth, teachers’ professional growth, and the development of preschool education. Therefore, it is of great practical significance to carry out research on the psychological capital of kindergarten teachers.

## Literature

Psychological capital was originally defined by Luthans et al. (2007b) as a positive psychological resource of individuals’ development and it is characterized by self-efficacy, optimism, hope, and resiliency. It possesses state-like qualities, which are more malleable than traits and more stable than states (Luthans et al., 2004). So psychological capital can not only have a significant impact on the health and growth of individuals by effectively regulating individuals’ psychology and emotions and then encouraging individuals to react to environmental changes with a positive attitude (Zhu et al., 2021); it can also have a significant impact on the competitiveness of organizations by effectively developed and managed.

The four characteristic structural elements of psychological capital were proposed by Luthans et al. (2007b) in the context of Western culture. Meanwhile, Luthans et al. (2007b) argued that cultural differences affect the set of resources that individuals acquire throughout their lifetime, which is consistent with “resource caravans” (Hobfoll, 2002). Because of the exploitable nature of psychological capital, different mental abilities are encouraged differently in cultures of individualism and collectivism. Based on positive organizational behavior, Ke et al. (2009) used grounded theory in the context of Chinese culture to explore a positive mentality that not only conformed to the connotation of psychological capital but also reflected the actual situation of employees in contemporary Chinese organizations, and they found that Chinese employees mainly use two types of psychological capital: task-oriented psychological capital and interpersonal-orientated psychological capital. The connotation of task-oriented psychological capital is basically similar to western psychological capital, which refers to the positive psychological state under the construction of the independent self. It emphasizes the positive psychological state of personal behavior toward the work goal, fearlessly and resolutely surviving difficulties and achieving the work goal. And it includes the spirit of enterprise and diligence, resiliency and perseverance, optimism and hope, and self-confidence and courage. Interpersonal-orientated psychological capital reflects the influence of the tradition of dependence on individuals in Chinese culture, which means that it is under the construction of the dependent self. The focus of individual behavior who own interpersonal-orientated psychological capital is to adjust their relationship with others, and tend to think of satisfying the expectations or requirements of others so as to maintain a harmonious positive psychological state of social relations. Interpersonal-orientated psychological capital includes toleration and forgiveness, modesty and prudence, thanksgiving and dedication, and respecting and courtesy (Ke et al., 2009).

According to the Job Demand-Resource Model (Job Demand-Resource Model, JD-R; Demerouti et al., 2001), continuous job demands drain employees’ energy and lead to burnout and physical harm. Conversely, the obtained work resources stimulate employees’ work motivation and produce good work results. Organizational expectations of employees in terms of child care and education, administrative affairs, and parent communication belong to the area of job demand and these expectations may cause stress to employees. How this stress ultimately affects an employee’s psychological capital depends on what kind of psychological capital the employee takes to respond to the organization’s psychological performance requirements. In addition, psychological capital falls under the heading of job resources. Studies have shown that psychological capital is an important resource for managing stressful situations and surroundings at work (Lazarus and Folkman, 1984). And workers with high psychological capital are more likely to persevere and get through difficulties in demanding work environments rather than give up (Luthans et al., 2004). Based on the above, the influence of psychological capital and different kind of psychological capital (task-oriented psychological capital and interpersonal-orientated psychological capital) becomes important and is a valuable issue to study.

Work engagement is a complete work state that consists of three aspects: vitality, dedication, and absorption. It is full of long-lasting, pleasant emotions and motivation (Schaufeli et al., 2002). According to The Conversation of Resource Theory (COR; Salanova et al., 2010), the accumulation of resources is the key driving force in motivating and maintaining individual behavior. If the resources invested by employees can be exchanged for greater returns, employees can develop an “acquisition spiral” mentality and invest more resources that they possess into work. In contrast, the reverse results in a “loss spiral” that maintains and protects current resources and exhibits job burnout (Kim and Kweon, 2020). Thus, job resources not only increase the risk of work burnout but also serve as a key predictor of work engagement (Demerouti et al., 2001; Schaufeli and Bakker, 2004). Numerous studies have demonstrated that psychological capital, used as an individual’s psychological resource, can explain the emergence of employee attitudes, behaviors, and performance (Avey et al., 2011; Bouckenooghe et al., 2019). Some studies have used work engagement as an important indicator to examine the impact of psychological capital on mental health. Recently, theoretical developments have revealed that psychological capital is significantly positively correlated with individuals’ work engagement, and increasing psychological capital promotes one’s work engagement (Wang and Luo, 2015; Ma et al., 2021; Guo et al., 2022). Such as, research by Firdaus (2019) found that employees’ psychological capital can positively influence their work engagement. Handayani and Damayanti (2020) further confirmed the above finding in a study of healthcare workers in Indonesia. By combing through relevant literature, it was found that psychological capital could mediate partnerships (Nigah et al., 2012), professional identity (Jin, 2015), and social support (Cheng and Gao, 2019). Specific to the two dimensions of psychological capital, task-oriented psychological capital and interpersonal-orientated psychological capital, Wu et al. (2012) found that the task-oriented and interpersonal-orientated psychological capital of primary and secondary school teachers both affect work engagement through the dual processes of energy replenishment and motivation, but the energy replenishment effect of interpersonal-orientated psychological capital was greater. Cheng and Gao (2019) also found that the eight sub-dimensions of psychological capital can significantly predict work engagement and its different dimensions. However, findings of Jin’s (2015) revealed that while resiliency and perseverance, optimism and hope, and self-confidence and courage in primary and secondary school teachers can positively predict work engagement and its various dimensions, modesty and prudence cannot significantly predict work engagement and its various dimensions. The results of Xiang (2022) demonstrated that, in contrast to tolerance, special education teachers’ thanksgiving and dedication and resiliency and perseverance were able to significantly predict work engagement.

All of the above studies are based on the Variable-Centered Approach. These contradictory results suggest that there can be intergroup differences among kindergarten teachers in terms of the level of psychological capital. Latent profile analysis (LPA) represents a good method of investigating this. It is a statistical method with a person-centered approach, which allows mixing between dimensions inside a variable. Using LPA, researchers can analyze the mixed population characteristics of categories and capture group inequalities that cannot be observed in variable-centered studies (Wang and Hanges, 2011). Thus, LPA can provide a scientific approach to identify the classification of kindergarten teachers’ psychological capital profiles, thereby helping researchers better understand individual differences. At present, there have been some studies to explore the potential profiles of psychological capital. But most of these studies have not taken preschool teachers as the research object, such as Bouckenooghe et al. (2019) focused on clerical staff, Zhu et al. (2021) rural left-behind children, and Chen et al. (2021) examined college students. And most of these studies were conducted in the western cultural background, without considering the situation of psychological capital under the cultural background of China. And there is also little research focus on the relationship between psychological capital and work engagement by using the latent profile method.

Therefore, this study focuses on kindergarten teachers and uses the method of LPA to explore the latent categories of kindergarten teachers’ psychological capital and the relationship between psychological capital and work engagement from the perspective of group heterogeneity. This study can provide a factual foundation for kindergarten teachers’ self-regulation and kindergarten administration.

## Materials and methods

### Participants and procedure

This study adopted a stratified random sampling method. Three representative regions were selected from the more developed, generally developed, and poor regions in Shaanxi Province. One county, one town, and one city were chosen for each region. Finally, a total of 27 towns, 9 counties, and three cities were randomly selected. A total of 2,825 questionnaires were distributed to kindergarten teachers and 2,790 valid questionnaires were finally recovered. The effective recovery rate of the questionnaire was 98.76%. Following institutional ethics approval, consent was obtained from the kindergarten principal to enter the kindergarten to distribute questionnaires. Before the test, participants were given informed consent. Also, participants were informed about the study and their right to refuse to complete the test. Then the trained postgraduates intensively illustrated the purpose of this study, the instructions for the questionnaire, and the precautions for participants. The questionnaire was carried out by means of unified organization, on-site distribution, centralized filling, and on-site recovery and took approximately 20 min to complete. More demographic information about the participants is presented in Table 1.

**Table 1:** Demographic information about the participants.

		Number	%
Region	The more developed	1,021	36.59%
	Generally developed	830	29.75%
	Poor	939	33.66%
Gender	Male	65	2.33%
	Female	2,725	97.67%
Teaching experience	≤5 years	1,651	59.17%
	6–10 years	667	23.91%
	11–15 years	227	8.14%
	16–20 years	142	5.09%
	≥21 years	103	3.69%
Kindergarten location	City	921	33.01%
	Country	935	33.66%
	Town	934	33.48%
Type of kindergarten	Public	1,406	50.39%
	Private	1,384	49.61%

### Measures

#### Psychological capital

Psychological capital was measured through the Chinese Indigenous Psychological Capital Scale (Ke et al., 2009). This 40-item scale has two dimensions, including task-oriented psychological capital and interpersonal-orientated psychological capital. And the items are slightly reworded to fit the kindergarten teaching context. In this scale, each dimension has four sub-dimensions, respectively. Task-oriented psychological capital includes: (1) Spirit of enterprise and diligence (five items, such as “I like to keep setting higher goals for myself”), (2) Resiliency and perseverance (five items, such as “I am a person who will never give up until the end”), (3) Optimism and hope (five items, such as “I feel that I have always been optimistic, and I hardly ever get depressed”), and (4) Self-confidence and courage (five items, such as “I am full of confidence in my ability to work”). Interpersonal-orientated psychological capital includes: (1) Toleration and forgiveness (five items, such as “I can make friends with almost any personality”), (2) Modesty and prudence (five items, such as “If I encounter problems that I do not understand, I will humbly ask my colleagues for help”), (3) Thanksgiving and dedication (five items, such as “I often think of people who helped me before”), and (4) Respecting and courtesy (five items, such as “I have almost no contradiction with my leader”). Chinese indigenous interpersonal content is included in this scale. And this scale has been used in a large number of previous studies in China with good reliability and validity. Items in this scale were scored on a six-point Likert scale, from 1 (strongly disagree) to 6 (strongly agree). The higher the score on the two dimensions, the higher the psychological capital level of kindergarten teachers. In the current study, Cronbach’s alphas were 0.934 (task-oriented psychological capital), 0.941 (interpersonal-orientated psychological capital), and 0.961 for the overall scale. The split-half reliability was 0.850 (see Table 2).

**Table 2:** Descriptive statistics and correlation coefficients (*N* = 2,790).

Variable	M ± SD	1	2	3	4	5	6	7
1. Task-oriented psychological capital	5.02 ± 0.81	–						
2. Interpersonal-orientated psychological capital	5.20 ± 0.70	0.75^**^	–					
3. Psychological capital	5.07 ± 0.63	0.95^**^	0.92^**^	–				
4. Vigor	4.31 ± 0.67	0.73^**^	0.73^**^	0.78^**^	–			
5. Dedication	4.39 ± 0.68	0.71^**^	0.70^**^	0.76^**^	0.90^**^	–		
6. Absorption	4.28 ± 0.68	0.69^**^	0.71^**^	0.74^**^	0.92^**^	0.90^**^	–	
7. Work engagement	4.31 ± 0.62	0.73^**^	0.74^**^	0.79^**^	0.97^**^	0.96^**^	0.97^**^	–

** *p* < 0.01.

#### Work engagement

The survey on work engagement of kindergarten teachers adopted the Utrecht Work Engagement Scale (UWES), which was designed by Schaufeli et al. (2002). Seventeen items are divided into three dimensions including vigor (e.g., I am energetic when I am working), dedication (e.g., I have a passion for early childhood education), and absorption (e.g., It makes me feel happy when I devote myself to my work). To fit the kindergarten teaching context, the 17 items in this scale were slightly reworded. And the 17 items were rated on a five-point Likert scale, ranging from 1 to 5 (1 = never, 5 = always). Higher scores indicated higher levels of work engagement. In this study, Cronbach’s alphas were 0.966 for the total scale, 0.910 for vigor, 0.914 for dedication, and 0.965 for absorption. The split-half reliability was 0.965.

### Statistical analysis

In this study, SPSS 26.0 and Mplus 8.2 were used for data statistics. First, this study was a cross-sectional study. Data were collected at the same time point and the three scales were all self-rated by participants. Consequently, there may be common methodological bias. To remove the risk of systematic error caused by self-reporting bias, Harman’s single-factor test was used to test the common method bias of the data. Second, Descriptive analyses and Pearson correlation analyses were conducted with basic SPSS functions to test the correlation between psychological capital and work engagement. Third, in order to identify the latent categorical variables to group the participants together in profiles and determine the optimal model, the LPA of psychological capital was carried out based on the two dimensions of psychological capital. The following parameters were considered as criteria to determine the optimum number of classes: the Akaike Information Criterion (AIC; Akaike, 1987), the Bayesian Information Criterion (BIC; Schwarz, 1978), the Sample Size-Adjusted Bayesian Information Criterion (SSA-BIC; Sclove, 1987), the Lo–Mendell–Rubin Likelihood Ratio Test (LMRLR; Lo et al., 2001), and the Bootstrap Likelihood Ratio Test (BLRT; McLachlan and Peel, 2004). The AIC, BIC, and SSA-BIC indices are descriptive, with lower values indicating a better model fit. The significant LMR and BLRT results (*p* < 0.05) indicate that the solution with k classes has a better fit to the data than a solution with k–1 classes (Nylund et al., 2007). We also used Entropy to assess the quality of the classifications, where a value higher than 0.80 suggests that the accuracy of the classification was over 90% (Lubke and Muthén, 2007). Fourth, this study examined the sample distribution in various latent profiles of psychological capital under demographic variables (i.e., type of kindergarten, kindergarten location, and teaching experience). Fifth, analyses were conducted to explore the relationship between psychological capital and work engagement from the perspective of potential profile, including one-way ANOVA and multinomial logistic regression analysis. The third analysis used Mplus 8.2, and the others used SPSS 26.0.

## Results

### Common method bias

Harman’s single-factor test was used to test the common method bias. There were five factors with an eigenvalue greater than 1, and the variance of the first factor was 33.28% (<40%). This indicated that there was no serious common method bias in the data.

### Descriptive statistics and correlation analysis of important variables

The descriptive statistics showed that the scores of total psychology capital and its two dimensions of kindergarten teachers were all higher than four points, while the scores of total work engagement and its three dimensions were all higher than three points. It indicated that the sample teachers in this survey generally own higher psychological capital and work engagement in their work. Specifically, the score of interpersonal-orientated psychological capital was higher than task-oriented psychological capital, and the order of the scores of each dimension of work engagement from high to low was dedication > vigor> absorption.

### Latent profile analysis

#### Model fitting

Based on the two dimensions of kindergarten teachers’ psychological capital, profile 1 to profile 6 were selected for a latent profile analysis. The results are shown in Table 3. The values of AIC, BIC, and SSA-BIC were getting smaller and smaller with additional profiles and the *p* values for BLRT were all statistically significant (*p* < 0.001). The entropy values of the six models were all greater than 0.7 while the three-profile, four-profile, five-profile, and six-profile models were above 0.8. This showed that the one-profile and the two-profile solution was not reliable compared to the latter four solutions. And the LMRLR for the six-profile model was not statistically significant (*p* > 0.5) while statistically significant for the 1-profile model to 5-profile model (*p* < 0.001), indicating that the six-profile solution was not superior to the five-profile solution and the latter one should be retained.

**Table 3:** Model fit indices of the latent profile analysis (*N* = 2,790).

Model	AIC	BIC	SSA-BIC	Entropy	LMRLR(p)	BLRT(p)	Profile Probability
1. Profile	11214.846	11238.581	11225.872				
2. Profiles	9770.737	9812.274	9790.033	0.726	0.000^***^	0.000^***^	0.698/0.302
3. Profiles	8968.236	9027.574	8995.801	0.801	0.000^***^	0.000^***^	0.463/0.105/0.432
4. Profiles	8623.197	8700.336	8659.031	0.831	0.048^*^	0.000^***^	0.016/0.401/0.149/0.433
5. Profiles	8504.462	8599.403	8548.565	0.855	0.036^*^	0.000^***^	0.001/0.396/0.019/0.157/0.425
6. Profiles	8380.352	8493.094	8432.725	0.870	0.234	0.000^***^	0.145/0.001/0.016/0.387/0.428/0.023

AIC, Akaike information criterion; BIC, Bayesian information criterion; SSA-BIC, sample size-adjusted Bayesian information criterion; LMRLR, Lo–Mendell–Rubin likelihood Rratio test; and BLRT, bootstrap likelihood ratio test.

^*^p < 0.05，^**^p < 0.01，^***^p < 0.001.

**Figure 1:**
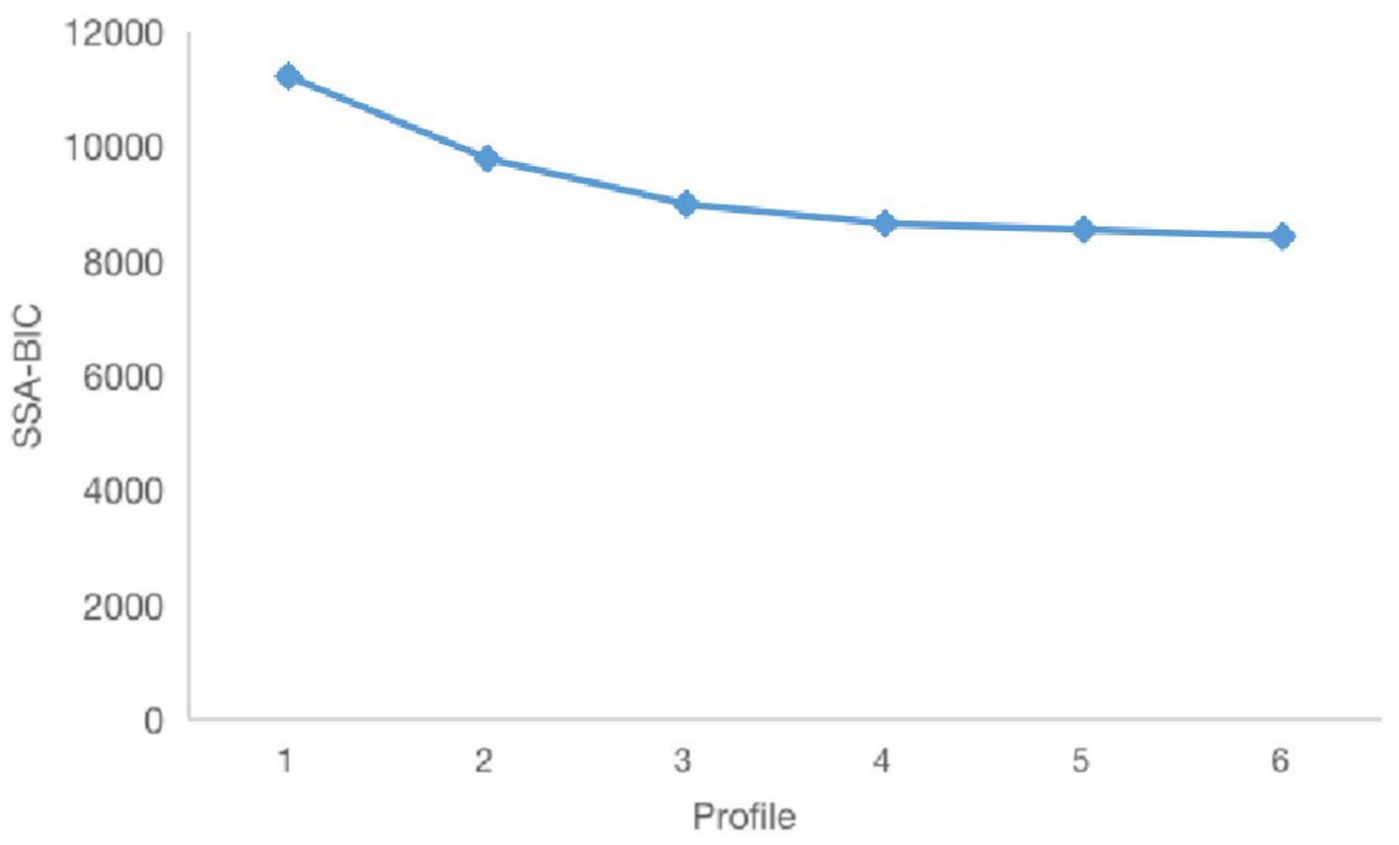
Elbow plot for sample size-adjusted Bayesian information criterion (SSA-BIC).

Figure 1 provides the trend of SSA-BIC value for models 1–6. It can be found that the value of SSA-BIC has an obvious inflection point when the model was classified into three latent profiles. It can be preliminarily determined that the 3-profile model is reliable. Meanwhile, as seen in Table 3, one profile’s probability was only 1.6% when the model was divided into four profiles. And one profile in the five-profile solution model consisted of only 1% of the sample while another 1.9%. All the above demonstrates that model with three latent profiles is reliable.

Table 4 presents Average Latent Class Probabilities for Most Likely Latent Class Membership (Row) by Latent Class (Column). As seen in Table 4, the average probabilities of class membership for three profiles were higher than 0.90, ranging from 90.2 to 92.3%.

**Table 4:** Most likely latent profile membership (row) by latent profile (column).

Profile	1 (%)	2 (%)	3 (%)
1	0.902	0.029	0.073
2	0.091	0.909	0.000
3	0.077	0.000	0.923

#### Description of the profiles

**Figure 2:**
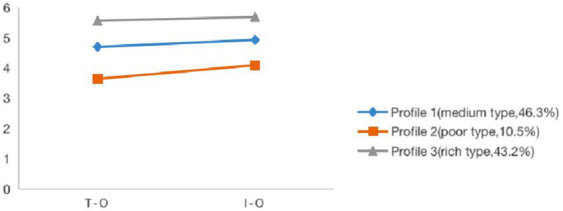
Mean scores in the dimensions of psychological capital for the three-profile solution. *T-O*, task-oriented; *I-O*, interpersonal-oriented.

Naming and analyzing the feature of latent profiles were based on the mean of task-oriented and interpersonal-orientated psychological capital. Figure 2 depicts a graphic description of the three-profile solution. The profile with the majority of the sample was named “medium type” (46.3%, M task-oriented = 4.70, M interpersonal-orientated = 4.93, Profile 1). Compared to the other two profiles, both task-oriented and interpersonal-orientated psychological capital in “medium type” were relatively high. The profile with the second highest proportion was named “rich type” (43.2%, M task-oriented = 5.56, M interpersonal-orientated = 5.69, Profile 3). And task-oriented and interpersonal-orientated psychological capital in this profile were both the highest in the sample. The profile with the smallest number was named “poor type” (10.5%, M task-oriented = 3.64, M interpersonal-orientated = 4.10, Profile 2), in which task-oriented and interpersonal-orientated psychological capital were both the lowest.

**Figure 3:**
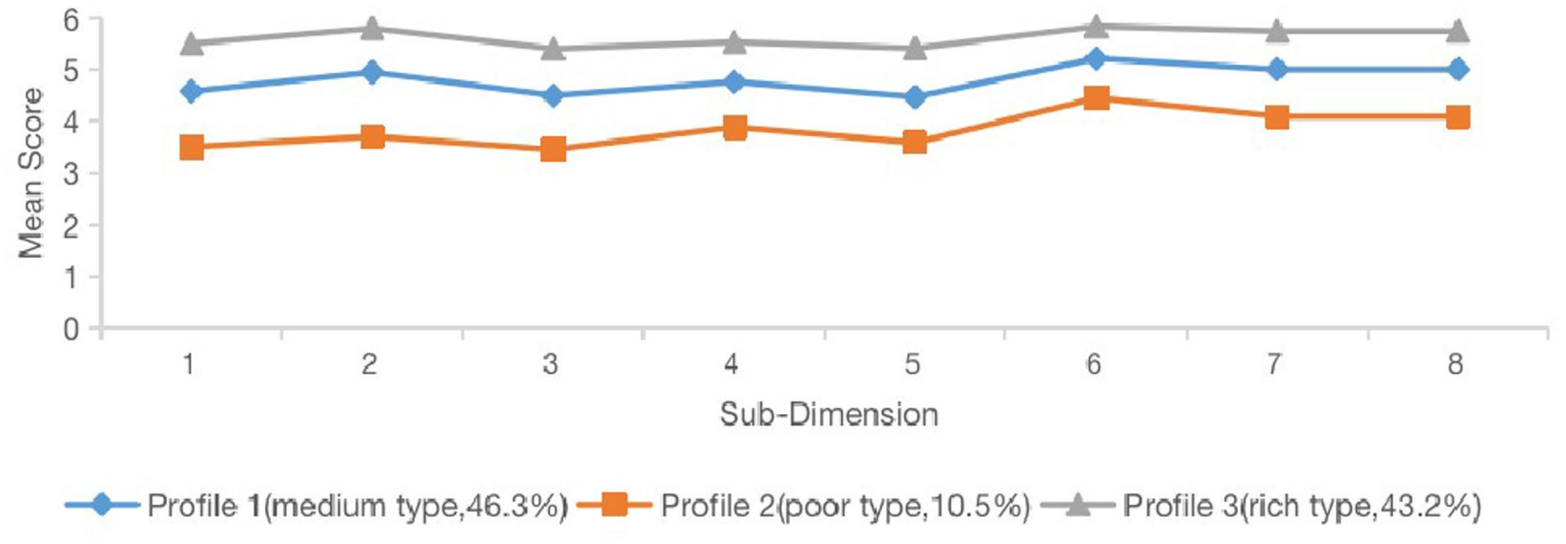
Mean scores in the sub-dimensions of psychological capital for the three-profile solution. 1 = Spirit of enterprise and diligence, 2 = Resiliency and perseverance, 3 = Optimism and hope, 4 = Self-confidence and courage; 5 = Toleration and forgiveness, 6 = Modesty and prudence, 7 = Thanksgiving and dedication, and 8 = Respecting and courtesy.

Figure 3 describes the mean of each sub-dimension of psychological capital of the three profiles in detail. As seen in Figure 3, “rich type” had the highest mean scores in each of the eight sub-dimensions among the three profiles, “medium type” has the second highest mean scores in each of the eight sub-dimensions, and the mean scores of each sub-dimension were at the lowest level in “poor type.”

#### Differential analysis of demographic variables among the latent profile types of kindergarten teachers’ psychological capital

Based on the results of the above analysis of the latent profiles of psychological capital, this study further explored the effects of type of kindergarten, kindergarten location, and teachers’ teaching experience on psychological capital. The results of the three latent profiles were used as dependent variables, and the type of kindergarten, kindergarten location, and teachers’ teaching experience indicators were used as independent variables. The chi-square test results are shown in Table 5. The results showed that the differences in the potential profiles of teachers’ psychological capital in the above-mentioned demographic variables were statistically significant. Compared to private kindergarten teachers, public kindergarten teachers tended to be more belong to “rich type,” while fewer “poor type” and “middle type.” There were more “rich type” and “middle type” among kindergarten teachers in city, with “rich type” making up the highest proportion and “poor type” the lowest. And from city to county to town, the proportion of “poor type” steadily declined while the proportion of “rich type” gradually rose. With the increasing teaching age, the “middle type” and the “poor type” both grew in a u-shape, whereas the “rich type” developed in an inverted u-shape. Compared with other teaching ages, the proportion of “poor type” and “middle type” teachers was the lowest and the proportion of “rich type” teachers was the highest when the teaching age was between 11 and 15 years. Additionally, the proportion of “rich type” teachers was lowest and the proportion of “poor type” and “middle type” teachers was at its highest when the teaching age was less than 5 years.

**Table 5:** Comparison among the different latent profiles of psychological capital in terms of differences regarding demographic variables.

Variable	Poor type	Medium type	Rich type	*χ* ^2^
**Type of kindergarten**				61.002^***^
Public	93 (6.70%)	614 (44.40%)	677 (48.90%)	
Private	201 (14.30%)	677 (48.20%)	528 (37.60%)	
**Kindergarten location**				278.803^***^
City	0 (0.00%)	437 (47.4%)	484 (52.6%)	
Country	107 (11.4%)	363 (38.3%)	465 (49.7%)	
Town	187 (20.0%)	491 (52.6%)	256 (27.4%)	
**Teaching experience**				40.661^***^
≤5 years	190 (11.5%)	811 (49.1%)	650 (39.3%)	
6–10 years	59 (8.8%)	306 (45.8%)	302 (45.2%)	
11–15 years	18 (7.9%)	74 (32.5%)	135 (59.4%)	
16–20 years	16 (11.2%)	55 (38.7%)	71 (50.00%)	
≥21 years	11 (10.6%)	45 (43.6%)	47 (45.6%)	

### Relationship between psychological capital and work engagement of kindergarten teachers

#### Single factor analysis for work engagement

With three latent profiles of psychological capital taken as independent variables and the total work engagement and each sub-dimension of work engagement used as dependent variables, a one-way ANOVA was performed. The results are shown in Table 6. It displayed that there were significant differences in mean scores of work engagement in terms of kindergarten teachers with different latent profiles of psychological capital (*F* = 1492.230, *p* < 0.001). LSD results revealed that the mean values differed significantly among the three profiles. And the differences between every two latent profiles were all statistically significant (*p* < 0.001), presenting the following situation: profile 3 (*M* = 4.77, SD = 0.33) > profile 1 (*M* = 4.09, SD = 0.45) > profile 2 (*M* = 3.41, SD = 0.66). In the sub-dimensions of psychological capital, the mean values of vigor (*F* = 1484.40, *p* < 0.001), dedication (*F* = 1290.03, *p* < 0.001), and absorption (*F* = 1180.49, *p* < 0.001) with three profiles were also significantly different. And the trend was profile 3 > profile 1 > profile 2.

**Table 6:** One-way ANOVA results of kindergarten teachers’ work engagement with different latent profile.

	Work engagement	Vigor	Dedication	Absorption
Profile 1 (Medium type)	4.09 ± 0.45	4.07 ± 0.47	4.16 ± 0.48	4.04 ± 0.48
Profile 2 (Poor type)	3.41 ± 0.66	3.36 ± 0.66	3.47 ± 0.71	3.40 ± 0.70
Profile 3 (Rich type)	4.77 ± 0.33	4.78 ± 0.35	4.82 ± 0.34	4.72 ± 0.39
*F*	1492.23^***^	1484.40^***^	1290.03^***^	1180.49^***^
LSD	Rich type > medium type ^***^	Rich type > medium type ^***^	Rich type > medium type ^***^	Rich type > medium type^***^
	Medium type > poor type^***^	Medium type > poor type^***^	Medium type > poor type^***^	Medium type > poor type^***^
	Rich type > poor type^***^	Rich type > poor type^***^	Rich type > poor type^***^	Rich type > poor type^***^

*** *p* < 0.001.

#### Multiple factors analysis

To further explore the relationship between psychological capital and work engagement, a one-way ANOVA was conducted with the three demographic variables including the type of kindergarten, kindergarten location, and teaching experience. The results showed that the mean scores of work engagement in the type of kindergarten (*t* = −8.88, *p* < 0.001), kindergarten location (*F* = 822.86^***^, *p* < 0.001), and teaching experience (*F* = 10.02^***^, *p* < 0.001) were all significantly different. In order to control the above three variables’ influences on psychological capital, a multinomial logistic regression analysis was conducted with the above three variables used as the first layer and the potential profile of psychological capital as the second layer. On the basis of results, the variance inflation factors were all less than 3, which indicates that there was no obvious collinearity among the variables. And the R-square change was 0.224 (as seen in Table 7) meant that psychological capital still had a positive impact on work engagement after the potential profile of psychological capital was added. Above all, it can be concluded that psychological capital is very important and it has a great impact on kindergarten teachers’ work engagement.

**Table 7:** Multinomial logistic regression analysis results of kindergarten teachers’ work engagement with different latent profile.

	Layer 1	Layer 2
(constant)	4.813^***^	4.078^***^
Type of kindergarten	−0.192^***^	−0.146^***^
Kindergarten location	0.808^***^	−0.11^***^
Teaching experience	1.808^***^	0.032^***^
Latent profile of psychological capital		0.316^***^
*F*	80.202^***^	303.66^***^
*R* ^2^	0.079	0.304
Adjusted *R*^2^	0.079	0.303
△*R*^2^	0.079	0.224

*** *p* < 0.001.

## Discussion

The results of latent profile analysis show that there is group heterogeneity in the psychological capital of kindergarten teachers, which is consistent with previous research findings (Bouckenooghe et al., 2019; Ferradás et al., 2019; Chen et al., 2021; Zhu et al., 2021). This study found that psychological capital could be divided into three types: “rich type,” “medium type,” and “poor type.” But previous studies such as Ferradás et al. (2019) classified psychological capital into seven categories, Bouckenooghe et al. (2019) divided psychological capital into six categories, and Chen et al. (2021) classified psychological capital into four categories.

This inconsistent result may be due to the following reasons. First, the subjects of study are different. This study focused on early childhood teachers. However, early research optioned clerical staff employed in different occupational sectors (Bouckenooghe et al., 2019), rural left-behind children (Zhu et al., 2021), college students (Chen et al., 2021), or teachers worked in different educational stages (Ferradás et al., 2019); Second, different studies used different measurement tools. Early studies mostly used Psychological Capital Questionnaire including efficacy, hope, optimism, and resilience four factors (PCQ-24, Luthans et al., 2007a,b; Bouckenooghe et al., 2019; Chen et al., 2021) or a questionnaire that was adjusted by PCQ-24 according to the needs of research (Ferradás et al., 2019; Zhu et al., 2021). This study used the Chinese Indigenous Psychological Capital Scale (Ke et al., 2009) which had 40 items and two dimensions measuring task-oriented and interpersonal-oriented psychological capital. Third, different cultures may have different effects on model classification. A study has shown that cultural traits play different roles in the mechanisms of psychological capital (Ke et al., 2009). And this result was supported by studies conducted in Spain (Ferradás et al., 2019) and Pakistan (Bouckenooghe et al., 2019). But this study was conducted in China.

In this study, the three latent profiles of kindergarten teachers’ psychological capital fit a variety of indices with reasonable accuracy. Among the three potential categories, the proportion of “poor type” was the lowest. This is in line with earlier research (Zhu et al., 2021). And the lowest scores were obtained by “poor type” kindergarten instructors in both the total task-oriented psychological capital and the total interpersonal-oriented psychological capital, as well as in each of their four sub-dimensions. Kindergarten teachers belonging to “poor type” are less able to provide psychological support and frequently not optimistic and hopeful when they face difficulties and failures in their work. And they are unable to think positively, sum up experiences and lessons, and work diligently in order to overcome obstacles (Cheng and Gao, 2019). Both the proportion of “medium type” and “rich type” kindergarten teachers was above 40%. And the scores were all at a relatively high level whether it came to total task-oriented psychological capital and its four sub-dimensions or total interpersonal-orientated psychological capital and its four sub-dimensions. It is clear that kindergarten teachers with higher psychological capital have higher personal resources. They have the capacity to handle stress and challenges at work with a positive attitude, daring to confront problems head-on and offer suggestions for the development of both individuals and organizations. Meanwhile, kindergarten teachers with higher psychological capital have a high level of loyalty to their organization, allowing them to manage interpersonal connections between themselves and other members of their organization because they have a high level of toleration and forgiveness, modesty and prudence, thanksgiving and dedication, and respecting and courtesy. All the above further explains The Conversation of Resource Theory (COR; Salanova et al., 2010). In general, the profile lines of the three categories in this research model showed a relatively consistent trend. However, a careful comparison of the item content revealed that in the “poor type,” the scores of the spirit of enterprise and diligence and optimism and hope were particularly low, while optimism and hope and toleration and forgiveness were the lowest in the “middle type” and “poor type.” It shows that all three types of preschool teachers need to improve their optimism and hope, preschool teachers with relatively high psychological capital may take their toleration and forgiveness seriously.

The results indicated that among the latent categories of psychological capital of kindergarten teachers, there were significant differences in the type of kindergarten, kindergarten location, and teaching experience. First, most of the public kindergarten teachers were “rich type,” whereas the majority of the private kindergarten teachers were “medium type” and “poor type.” This may be due to the fact that public kindergartens have access to resources that private kindergartens do not, such as advanced training, professional title evaluation, and status treatment. These resource components help teachers build their psychological capital obstacles (Cheng and Gao, 2019). Second, there were a number of “poor type” and “medium type” town teachers. With the decline of the economic development level of the kindergarten location, the proportion of “poor type” teachers steadily increased, and the proportion of “rich type” teachers gradually decreased. This may be due to that those kindergarten teachers in town have a limited network and limited access to the most recent educational ideas. Thus, they are less optimistic and hopeful in education and teaching (Cheng and Gao, 2019) Third, with the increasing teaching experience, “rich type” took on an inverted u-shape, and “medium type” and “poor type” also took on a u-shape overall. This means that the teaching years between 11 and 15 years have the highest proportion of “rich type” and the lowest proportion of “poor type” and “medium type.” The results basically agree with the findings of Qin and Yan (2007). Usually, the majority of kindergarten teachers between the 11 and 15 teaching ages have evolved into the foundation of their families, their communities, and society as a whole. As a result of their increased responsibilities, they have sufficient motivation to make some achievements in their careers. Meanwhile, kindergarten teachers’ abilities at work and interpersonal skills grow as their age and teaching experience increase. Therefore, special attention and support should be paid to kindergarten teachers with poor psychological capital, especially those in private kindergartens, towns, and newly recruited ones.

This study revealed that there were significant differences in the work engagement of teachers with different psychological capital types. The “rich type” teachers had the highest scores in terms of total work engagement, vigor, dedication, and absorption than the other two types, while the “poor type” teachers received the lowest scores. After controlling the variables of the type of kindergarten, kindergarten location, and teachers’ teaching age, the latent category of psychological capital of kindergarten teachers still had a substantial impact on work engagement. It implies that psychological capital is an essential factor affecting work engagement. According to the Conservation of Resources Theory (Hobfoll, 2011; Wu et al., 2012), in order to manage the negative effects of various stressors, individuals must mobilize their positive resources. However, poor people who own few resources may lack the necessary reserves to manage stress. In addition, they are more easily to accelerate their loss of resources and enter loss spirals by exhausting their reserves. Kindergarten teachers with low psychological capital usually have limited positive psychological resources and cannot effectively address the risks and crises they encounter; consequently, they develop relatively more work engagement. In contrast, individuals with certain good resources (such as optimism and hope) have the ability to access other resources, and their good resources can also generate additional resource increments (Firdaus, 2019). This means that these individuals have sufficient psychological capital to address various risks and crises. Thus, kindergarten teachers with high psychological capital are more likely to engage in their work.

### Implications

In terms of theory, first, this study explored how the potential type of kindergarten teacher’s psychological capital affects their work engagement from the perspective of potential profile analysis. And it found that kindergarten teachers with higher scores of psychological capital in the two dimensions of transactional and interpersonal psychological capital also had higher scores of work engagement. Establishing this link strengthens the role of psychological capital on work engagement, which is conducive to enriching the previous variable-centered research on psychological capital. Second, this study focused on preschool teachers and used a measurement tool of psychological capital with rich Chinese cultural connotations. Luthans and Youssef-Morgan (2017) ever highlighted the importance of analyzing psychological capital profiles in different contexts. Additionally, Dawkins et al. (2013) pointed out that psychological capital studies have been neglecting the importance of examining an individual’s psychological capital profile in different environments. In view of this, focusing on the group of kindergarten teachers in the context of Chinese culture is conducive to supplementing the previous research on the psychological capital of other groups.

In terms of practice, this study found that the type of psychological capital with a lower level of task-oriented and interpersonal-orientated psychological capital also had a lower level of work engagement. In conformance with the Conservation of Resources theory from Hobfoll (1989), the availability of resources (e.g., efficacy) drives the individual to a rising spiral of acquisition, development, and preservation of new resources (e.g., resilience, optimism, and hope). Interventions may benefit from this caravan action of resources (Hobfoll, 2002) to strengthen and enhance kindergarten teachers’ psychological capital overall.

## Conclusion

The current study conducted an LPA on inclusion in the Chinese context as an initial exploration into the profiles of psychological capital in a non-Western context. Using the 40-item Chinese Indigenous Psychological Capital Scale that fits well with the definition of psychological capital comprising task-oriented psychological capital and interpersonal-orientated psychological capital in the Chinese context, the results identified three psychology capital profiles among Chinese kindergarten teachers: (1) “rich type” (43.2%), (2) “medium type” (46.3%), and (3) “poor type” (10.5%). The results suggested that after controlling demographic variables such as the type of kindergarten, the location of kindergarten, and the teaching experience of teachers, different types of psychological capital still had a significant impact on work engagement. For this study, evidence was given to raise awareness of kindergarten teachers’ psychological capital in the organization and develop policies to strengthen the psychological capital in order to promote kindergarten teachers’ work engagement.

## Limitations

This study has several limitations that need to be highlighted. First, the sample was limited to areas in Shaanxi Province. Future research should consider the whole country in order to provide a more solid theoretical foundation for kindergartens to improve the mining and cultivation of the psychological capital of kindergarten teachers. Second, similar to other empirical studies, this study used cross-sectional data at a certain time point, which did not clarify the causality of variables. Future work may examine this relationship with longitudinal designs. Third, the self-report scales used in this study to collect data may affect the accuracy of the information obtained. Future work should combine questionnaires, deep interviews, and field observations to enhance the quality of the pertinent data.

## Data availability statement

The raw data supporting the conclusions of this article will be made available by the author, without undue reservation.

## Ethics statement

All procedures performed in studies involving human participants were reviewed and approved by Beijing Normal University, in accordance with the ethical standards of the institutional and/or national research committee and with the 1964 Helsinki declaration and its later amendments or comparable ethical standards. The patients/participants provided their written informed consent to participate in this study.

## Author contributions

YG designed, collected questionnaires, and wrote the original draft. YY wrote the original draft and funded the project. XL revised and edited the paper. All authors contributed to manuscript revision, read, and approved the submitted version.

## Funding

This study was supported by the Youth Project of the Ministry of Education of the People’s Republic of China (EGA220544).

## Conflict of interest

The authors declare that the research was conducted in the absence of any commercial or financial relationships that could be construed as a potential conflict of interest.

## Publisher’s note

All claims expressed in this article are solely those of the authors and do not necessarily represent those of their affiliated organizations, or those of the publisher, the editors and the reviewers. Any product that may be evaluated in this article, or claim that may be made by its manufacturer, is not guaranteed or endorsed by the publisher.
